# Design and Synthesis of a Fluorescent Probe with a Large Stokes Shift for Detecting Thiophenols and Its Application in Water Samples and Living Cells

**DOI:** 10.3390/molecules24020375

**Published:** 2019-01-21

**Authors:** Hua Liu, Chuanlong Guo, Shuju Guo, Lijun Wang, Dayong Shi

**Affiliations:** 1 CAS Key Laboratory of Experimental Marine Biology, Institute of Oceanology, Chinese Academy of Sciences, Qingdao 266071, China; baihualin55100@sina.cn (H.L.); gcl_cpu@126.com (C.G.); guoshuju@qdio.ac.cn (S.G.); 2 Laboratory for Marine Drugs and Bioproducts, Qingdao National Laboratory for Marine Science and Technology, Qingdao 266237, China; 3 Center for Ocean Mega-Science, Chinese Academy of Sciences, 7 Nanhai Road, Qingdao 266071, China; 4 University of Chinese Academy of Sciences, Beijing 100049, China; 5 Department of Pharmacy, College of Chemical Engineering, Qingdao University of Science and Technology, Qingdao 266042, China

**Keywords:** fluorescent probe, thiophenol, water samples, chalcone, living cells

## Abstract

A turn-on florescent probe (probe-KCP) was developed for highly selective detection of thiophenols based on a donor-excited photo-induced electron transfer mechanism. Herein, the synthesis of the probe, a chalcone derivative, through a simple straightforward combination of a carbazole-chalcone fluorophore with a 2,4-dinitrophenyl functional group. In a kinetic study of the probe-KCP for thiophenols, the probe displayed a short response time (~30 min) and significant fluorescence enhancement. In selection and competition experiments, the probe-KCP exhibited excellent selectivity for thiophenols over glutathione (GSH), cysteine (Cys), sodium hydrosulfide (NaSH), and ethanethiol (C_2_H_5_SH) in addition to common anions and metal ions. Using the designed probe, we successfully monitored and quantified thiophenols, which are highly toxic. This turn-on fluorescence probe features a remarkably large Stokes shift (130 nm) and a short response time (30 min), and it is highly selective and sensitive (~160-fold) in the detection of thiophenols, with marked fluorescence in the presence of thiophenols. probe-KCP responds to thiophenols with a good range of linearity (0–15 μM) and a detection limit of 28 nM (R^2^ = 0.9946) over other tested species mentioned including aliphatic thiols, thiophenol analogues, common anions, and metal ions. The potential applications of this carbazole-chalcone fluorescent probe was successfully used to determine of thiophenols in real water samples and living cells with good performance and low cytotoxicity. Therefore, this probe has great potential application in environment and biological samples.

## 1. Introduction

Thiophenols are small molecules that serve as essential raw materials in the synthetic chemical industry. For example: they are used in the preparation of pesticides, polymers, and pharmaceuticals [1,2]. However, thiophenols are also pollutants which are highly toxic to organisms, including humans, where thiophenol toxicity can have adverse physiological and psychological effects. Prolonged exposure to thiophenols can cause damage to the central nervous system, breathing difficulties, muscle weakness, paralysis of the limbs, a coma, and even death [3,4]. Previous research reported that the median lethal dose of thiophenols to fish and mice was 0.01–0.4 mmol/L and 46.2 mg/kg^3^, respectively. Thiophenols are considered a priority pollutant by the U.S. Environmental Protection Agency (EPA waste code: P014) [5,6]. There is an urgent need for the development of a rapid, selective, and sensitive method for monitoring of thiophenols in environmental samples and biological samples.

There are various traditional methods such as high-performance liquid chromatography, gas chromatography, and nonlinear spectroscopy, as well as nanomaterial-based sensors, available for the detection of thiophenols [7,8,9,10,11]. However, most of these can detect thiophenols only in vitro. Fluorescent probe-based methods for the detection of thiophenols have many advantages [12,13,14,15,16,17,18,19,20]. These include high sensitivity and high selectivity, real-time bio-imaging, operational simplicity, and short response times, all of which are important factors in the detection of thiophenols. The study of small molecule fluorescent probes has attracted more and more attention [21]., However, many probes are affected by interference from glutathione (GSH), cysteine (Cys), and homocysteine (Hcy) which have similar chemical structures with thiophenols, or probes have a narrow Stokes shift (<60 nm) which is detrimental for biological applications due to excitation backscattering effects [22,23,24,25,26,27]. Designing a fluorescent probe that can distinguish between aliphatic thiols and aromatic thiophenols in living cells is a huge challenge. 

The biological activities of chalcone derivatives are well known [28,29,30,31,32,33]. These derivatives also exhibit some excellent optical properties. Dyes based on chalcone derivatives are characterized by large diversity and flexible modes of synthesis, intramolecular charge transfer, large Stoke shifts, bright fluorescence, and a high quantum yield [34,35]. Therefore, chalcone derivatives have attracted intense attention in recent years. In the present study, a turn-on fluorescent probe-KCP, based on a donor-excited photo-induced electron transfer mechanism was designed and applied to detect thiophenols. The probe-KCP was synthesized through a simple straightforward combination of a chalcone unit, which served as a fluorescent chromophore, and a 2,4-dinitrophenyl moiety, which acted as a fluorescent quenching unit. As expected, the designed probe shown pronounced selectivity for thiophenols over common metal ions, anions, amino acids and aliphatic thiols. The probe-KCP was substantially nonfluorescent due to the strong quenching effect of the 2,4-dinitrophenyl group. Thus, the probe-KCP has excellent detection abilities for thiophenols and possesses marked fluorescence intensity enhancement (~160-fold). It also has a conspicuously large Stokes shift (~130 nm). Therefore, the probe has been successfully applied for the quantitative detection of thiophenols in real water samples and fluorescent imaging of thiophenols in living cells.

## 2. Results and Discussion

### 2.1. Synthesis of the Probe-KCP

**Scheme 1:**
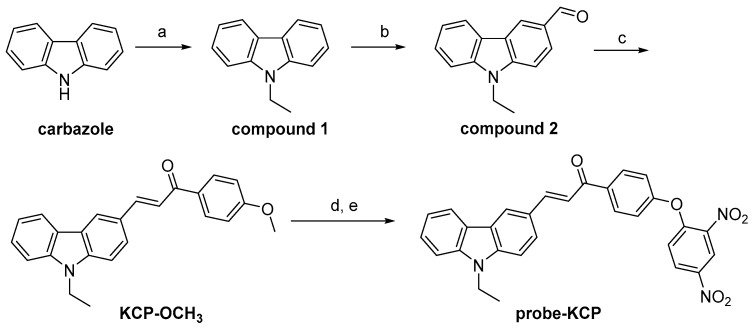
The synthetic routes and the chemical structures of the probe-KCP. *Reagents and conditions*: (**a**) NaH, DMF, bromoethane; (**b**) POCl_3_, DMF, 90 °C; (**c**) C_2_H_5_OH, C_2_H_5_ONa, 4′-methoxy-acetophenone; (**d**) BBr_3_, DCM, −78 °C; (**e**) 2,4-dinitrofluorobenzene, Cs_2_CO_3_, DCM, 30 °C.

The synthesis procedure of the target chalcone fluorescent probe-KCP for the detection of thiophenols is outlined in Scheme 1. The probe-KCP was synthesized with chalcones as a strong fluorophore linked to a 2,4-dinitrophenyl group acted simultaneously as recognition unit and a fluorophore quencher. KCP-OCH_3_ was reacted with boron tribromide in dry dichloromethane and cooled to −78 °C. The crude product was conveniently converted to the probe-KCP in dichloromethane at room temperature through a straightforward reaction with 2,4-dinitro-fluorobenzene. The structure of the probe was characterized by ^1^H-NMR, ^13^C-NMR, and MS spectra. The experimental characterization data are given in the Electronic Supplementary Information.

### 2.2. The Absorption and Emission Spectroscopic Properties of the Probe-KCP

**Figure 1:**
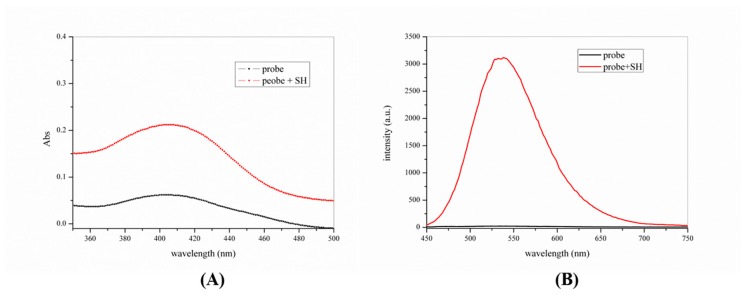
(**A**) Absorption spectra and (**B**) fluorescence spectra of probe-KCP (10 μM) in the presence of 100 μM 4-methoxythiophenol (red) in DMSO/PBS (1:1, *v*/*v*, 20 mM, pH = 7.4). *λ*_ex_ = 410 nm and *λ*_em_ = 540 nm.

To determine the fluorescence properties of the probe-KCP for thiophenols, we first examined the absorption and fluorescent spectra of probe. The reaction response time of the probe-KCP for thiophenols was investigated in DMSO/PBS (1:1, *v*/*v*, 20 mM, pH = 7.4). As shown in Figure 1, the absorption spectra of the probe (10 µM) solution displayed one band at 406 nm only after the addition of 4-methoxythiophenol (100 µM). The absorption maximum at 403 nm (*Φ* = 0.027, ε = 6.2 × 10^3^ M^−1^ cm^−1^) displayed a slight red shift 3 nm to 406 nm (*Φ* = 0.19; ε = 2.12 × 10^4^ M^−1^ cm^−1^). In the fluorescence spectra, significant fluorescence enhancement (~160-fold) was observed at 540 nm after 4-methoxy-thiophenol was added to the DMSO/PBS solution containing the probe-KCP (10 μM) and incubated for 30 min at 37 °C. The solution of the probe-KCP alone exhibited weak fluorescence intensity, with excitation at 410 nm. After the addition of 4-methoxythiophenol to the probe-KCP solution, it emitted relatively a strong fluorescent signal at 540 nm and an absorption maximum at 410 nm. The addition of 4-methoxythiophenol resulted in obvious green fluorescence from the probe-KCP solution when it was exposed to daylight or to a hand-held UV lamp. (Figure S8). These results demonstrated that the probe-KCP is a promising fast (30 min) turn-on fluorescence probe, with a large Stokes shift (130 nm) for the detection of thiophenols.

**Figure 2:**
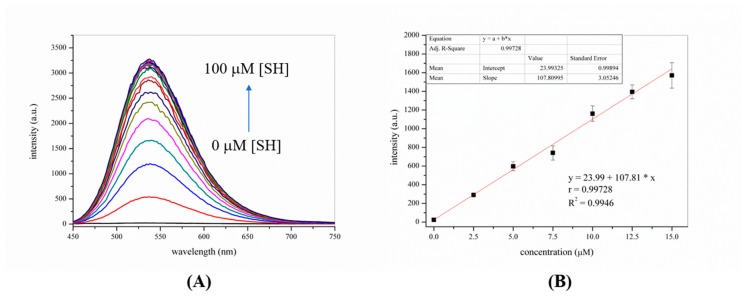
(**A**) Fluorescence spectra of 10 μM probe-KCP upon the addition of 4-methoxy thiophenol (0–100 μM); (**B**) The linearity of the probe-KCP fluorescence intensity versus thiophenol concentration (0–15 μM). *λ*_ex_ = 410 nm and *λ*_em_ = 540 nm. Relative standard deviation (RSD), *n* = 3.

To further investigate the sensitivity of the probe-KCP for thiophenols, the fluorescence properties of the probe in the presence of different concentrations of thiophenols were investigated. The probe-KCP detected thiophenols in DMSO/PBS (*v*/*v* =1:1, 20 mM, pH = 7.4) solvent at 37 °C. The probe-KCP (10 μM) on its own showed a maximum absorption peak at 403 nm and almost no fluorescence. However, when 4-methoxythiophenol was added to the probe solution, the maximum absorption peak red-shifted to 406 nm. As shown in Figure 2, the fluorescence intensity was proportional to the concentration of thiophenol and showed a rapid growth trend. When the concentration of 4-methoxythiophenol reached 60 μM, the fluorescence intensity was enhanced by about 160-fold and no change in the fluorescence at subsequent concentrations. An excellent linearly dependent (R^2^ = 0.9946) between the enhancement of fluorescence intensity and the concentration of 4-methoxythiophenol (0 μM to 15 μM), thereby indicating that the probe-KCP had the potential to be used for quantitatively monitoring thiophenols. The detection limit of the samples was calculated using the following equation:

LD = 3σ/k(1)

where σ is the standard deviation of a blank measurement, and k is the slope.

The kinetic study showed that the fluorescence intensity of the probe-KCP solution increased significantly 30 min after adding 4-methoxythiophenol at 37 °C and that the fluorescence intensity did not change substantially after 30 min (Figures S6 and S7). The results pointed to the potential use of the probe for real-time monitoring of thiophenols.

### 2.3. The Effect of pH on the Probe

The pH of a system can sometimes exert a marked effect on the fluorescence intensity. We tested the response of the probe-KCP to 4-methoxythiophenol over a wide pH range from 1 to 10. The influence of pH on the fluorescence spectra of the probe-KCP to 4-methoxythiophenol was tested. At a pH of <6, no significant changes in the fluorescence intensity of the probe-KCP solution were observed at 540 nm, and similar results were observed when the pH exceeded 10.0. However, at a pH of >6, the fluorescence intensity of the probe-KCP (10 μM) decreased significantly in the presence of 4-methoxythiophenol at a concentration of 100 μM. As shown in Figure S2, the probe-KCP functioned at 540 nm in a pH range from 7.0 to 10.0. The fluorescent intensity of the probe-KCP (10 μM) was stable over a wide pH range, which indicated that the probe was stable in the pH range of physiological conditions. Thus, in subsequent live cell imaging studies, a pH of 7.4 was selected as optimal for DMSO/PBS buffer (1:1, *v*/*v*, 20 mM).

### 2.4. Sensitivity and Selectivity of the Probe for Thiophenols

**Figure 3:**
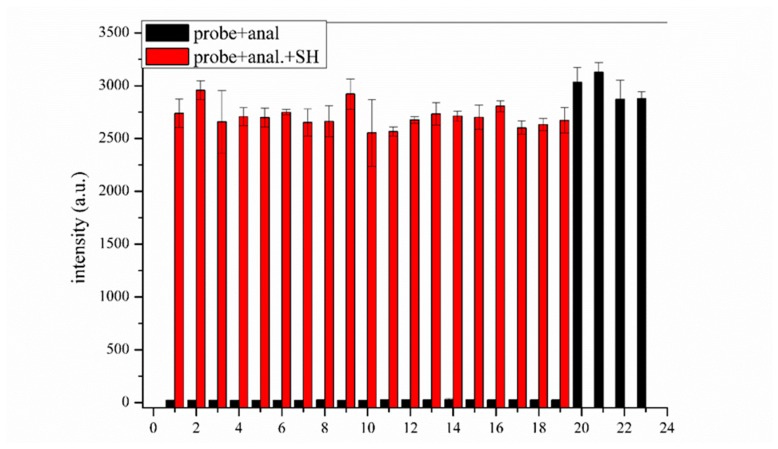
The fluorescence intensity at 540 nm (*λ*_ex_ = 410 nm) of probe-KCP (10 μM) in the presence of 100 μM of various analytes in DMSO/PBS buffer (1:1, *v*/*v*, 20 mM, pH 7.4) at 37 °C prior to (black bars) and after (red bars) addition of 100 μM 4-methoxythiophenol to the individual probe/analyte solution. (1) blank, (2) GSH,(3) Cys, (4) Hcy, (5) Ala, (6) Ser, (7) NaSH, (8) C_2_H_5_SH, (9) Ph-OH, (10) Ph-NH_2_, (11) NaCl, (12) FeCl_2_, (13) KCl, (14) CaCl_2_, (15) FeCl_3_, (16) MgSO_4_, (17) NaNO_3_, (18) KF, (19) NaHCO_3_, (20) 4-toluenethiol, (21) 4-methoxythiophenol, (22) 4-aminothiophenol, (23) 4-fluorothiophenol. Data were recorded 30 min after addition of analytes/ thiophenols with excitation at 410 nm and emission at 540 nm. Error bars are relative standard deviations (RSD), *n*=3.

Next, the spectral responses of the probe-KCP toward various thiophenols, thiols, amino acids, other potentially interfering substances, common anions and metal ions were investigated by fluorescence spectroscopy. As shown in Figures S3 and S4, only the introduction of thiophenols such as *p*-CH_3_−C_6_H_4_SH, *p*-NH_2_−C_6_H_4_SH, *p*-F−C_6_H_4_SH and *p*-CH_3_O−C_6_H_4_SH to the probe-KCP solution induced a significantly enhanced the fluorescence intensity. Reactivity comparison: p-CH_3_O−C_6_H_4_SH > *p*-F−C_6_H_4_SH > *p*-NH_2_−C_6_H_4_SH > *p*-CH_3_−C_6_H_4_SH. However, the addition of other biologically relevant analytes resulted in no change in the fluorescence intensity of the compounds in the probe-KCP solution. These results clearly suggested that the probe-KCP was particularly selective for thiophenols over other interfering species. In other words, the probe-KCP displayed high selectivity for thiophenols (4-fluorothiophenol, 4-toluenethiol, 4-methoxythiophenol, and 4-aminothiophenol). As shown in Figure 3, it is worth noting that there was no significant fluorescence signal change after the probe reacted with the other potentially interfering substances interferences (10 eq.), such as mercaptan, nucleophilic species, and metal ions in the DMSO/PBS buffer (1:1, *v*/*v*, 20 mM, pH = 7.4) at 37 °C for 30 min. These results showed that the probe-KCP could be used to monitor thiophenols in the presence of competitive interference from other substances.

### 2.5. Plausible Mechanism of Detection of Thiophenols

To determine a plausible mechanism for the detection of thiophenols, ^1^H-NMR, ^13^C-NMR, and mass spectrometry (Figures S1 and S9) were employed to monitor the reaction of the probe-KCP with 4-methoxythiophenol. Two products (probe-OH and S-NO_2_) were isolated and their structural characterizations are shown in Figure S1. 

**Figure 4:**
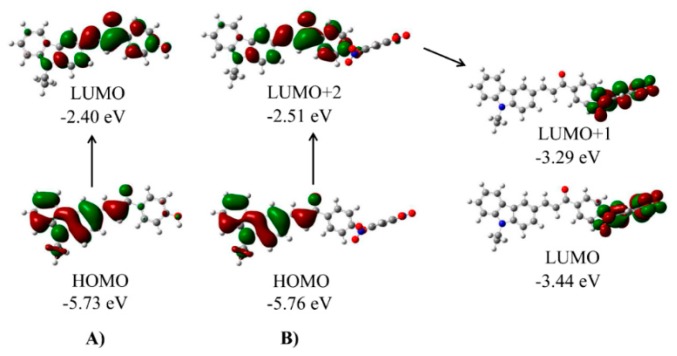
(**A**) Frontier molecular orbital plots of dye probe-OH (CPCM model); (**B**) Frontier molecular orbital plots of probe-KCP (CPCM model). The fluorescence emission of probe-KCP moieties is quenched by d-PET.

To better understand the reaction mechanism of the probe-KCP with thiophenols, density functional theory was applied using the Gaussian 09 program (Figure 4 and Figure S10). As shown in Figure 4A, for the probe-OH moiety, electron clouds on both HOMO and LUMO essentially resided on its entire skeleton. By contrast, in the case of the probe-KCP (Figure 4B), the electron clouds on the HOMO and LUMO+2 were mainly distributed in the electron-donating moiety of the probe-OH. However, those on the LUMO and LUMO+1 were primarily located on the electron-withdrawing 2,4-dinitrophenyl group. These findings indicated that the probe-KCP could bear efficient electron transfer from the moiety of the probe-OH to the 2,4-dinitrophenyl moiety, resulting in weak or no fluorescence of the probe. After the addition of 4-methoxythiophenol, the electron transfer from the moiety of the probe-OH to the 2,4-dinitrophenyl was blocked following the elimination of the 2,4-dinitrophenyl group. The resulting almost complete overlap of electrons on the transition orbitals may induce strong fluorescence emission from the moiety of the probe-OH.

### 2.6. Measurements of Thiophenols in Water Samples

To evaluate whether the probe could directly detect thiophenols in a real environmental sample, the probe was tested in experiments involving deionized water, artificial seawater, and tap water samples. The water samples were spiked with different concentrations of 4-methoxythiophenol and then incubated with the probe for 30 min at 37 °C. 

**Figure 5:**
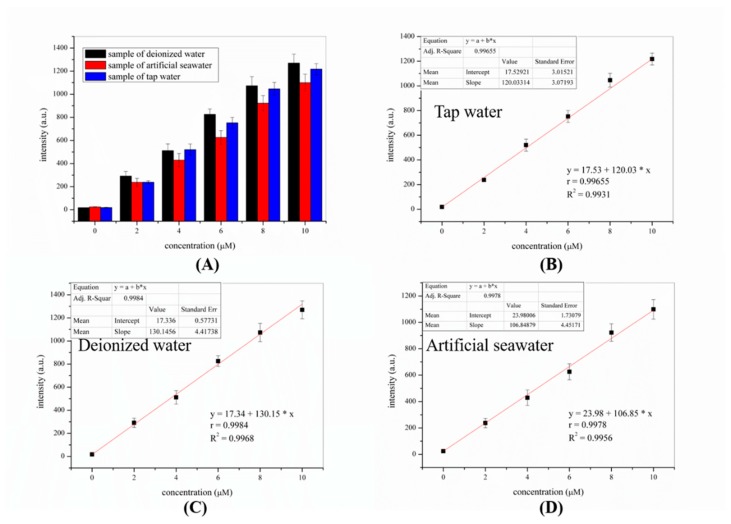
A linear calibration curve between the fluorescence intensities of probe-KCP and concentration of 4-methoxythiophenol in the range of 0–10 μM in real water samples. (**A**) Fluorescent detection of different concentrations of 4-methoxythiophenol in “tap water”, “deionized water”, and “artificial water” by probe-KCP (10 μM) in PBS buffer (20 mM) with 50% DMSO; (**B**–**D**) Linear of fluorescence intensity changes of probe-KCP against the of 4-methoxythiophenol for real water sample.

As shown in Figure 5, the experimental results showed that the fluorescence intensity of the probe increased in proportion to the concentration of 4-methoxythiophenol added and that the recovery of 4-methoxythiophenol was 92–107%. This finding indicated that the probe was capable of detecting thiophenols in real water samples. The results shown in Table 1 was reported as the mean ± standard deviation of triplicate experiments for thiophenol spiked from 0 to 10 μM.

### 2.7. Determination of the Potential Cytotoxicity of the Probe

To evaluate the potential cytotoxicity of the probe before applying it in live cell imaging, we performed an MTT assay in A549 cells. Different concentrations of the probe-KCP (0, 2.5, 5, 10, 20, and 50 μM) were added to the A549 cells and incubated for 24 h. As shown in Figure S5, the probe-KCP was nontoxic to the cultured cells under the experimental conditions, which demonstrated that the probe was safe and suitable for cell imaging.

### 2.8. Cell Imaging

**Figure 6:**
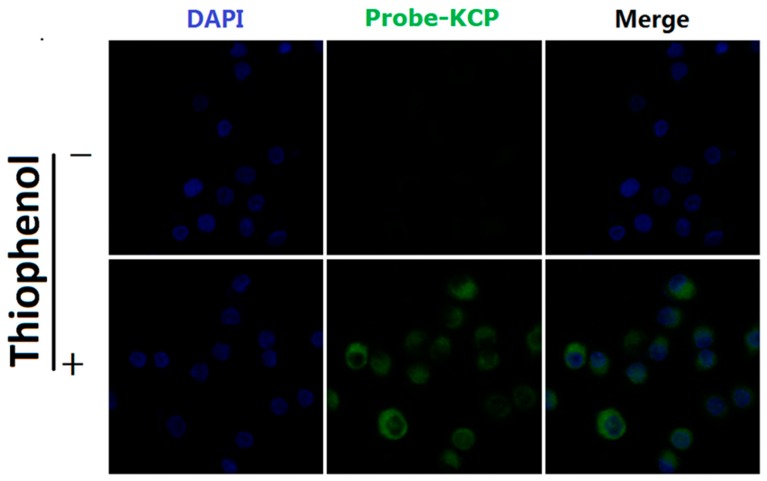
Imaging of 4-methoxythiophenol in living A549 cells using the probe-KCP. The A549 cells were pretreated with 4-methoxythiophenol (300 μM) for 30 min at 37 °C and then incubated with the probe-KCP (10 μM) for 30 min at 37 °C.

Given the favorable properties of the probe-KCP (e.g., high selectivity, excellent sensitivity, a short response time, and lack of cytotoxicity) in terms of the detection of thiophenols in vitro in DMSO/PBS buffer (1:1, *v*/*v*, 20 mM, pH = 7.4), we tested the potential applications of the probe-KCP in live cell imaging assays. The A549 cells were treated with the probe-KCP and then observed under a confocal fluorescence microscope. As shown in Figure 6, the probe-KCP (10 μM) was incubated with the A549 cells for 30 min at 37 °C. Almost no intracellular fluorescence signals were observed in the green channel by CLSM. However, following the addition of 300 μM 4-methoxythiophenol to the probe-KCP solution and incubation with the A549 cells for 30 min, green fluorescence signals were observed in the green channel. These findings indicated that the probe-KCP could be employed to detect thiophenols in A549 cells via CLSM. Thus, the designed probe-KCP is cell membrane permeable and can detect thiophenols with obvious fluorescence signals in living cells.

## 3. Experimental

### 3.1. Starting Materials and Instruments

Carbazole (CAS No. 86-74-8, 98%), bromoethane (CAS No. 74-96-4, 98%), 4′-methoxy-acetophenone (CAS No. 100-06-1, 99%), 2,4-dinitrofluorobenzene (CAS No. 70-34-8, 99%), boron tribromide (CAS No. 10294-33-4), and cesium carbonate (CAS No. 534-17-8, 99%) were obtained commercially and used without further purification. Other reagents (phosphorus oxychloride, ethanol, ethyl acetate, petroleum ether, dichloromethane, sodium bicarbonate, and anhydrous sodium sulfate) were of commercial analytical grade and were used without further purification. Silica gel (200−300 mesh, Qingdao Makall Group Co., Ltd., Qingdao, China) was used in silica gel column chromatography. The fins salt was obtained commercially (Qingdao Haike General Sea Salt Co., Ltd., Qingdao, China). The pH was determined by a pH meter (LEICI, Shanghai, China). UV-vis spectra were measured on a UV-Vis spectrophotometer (Agilent Technologies, Waldbronn, Germany). Fluorescence spectra were recorded on a Tecan Infinite M1000 Pro Microplate Reader (Tecan, Männedorf, Switzerland). ^1^H-NMR and ^13^C-NMR spectra were recorded on a Bruker Avance III^TM^ 500 MHz NB Digital NMR spectrometer (Bruker-Biospin, Billerica, MA, USA) in CDCl_3_ or DMSO-*d*_6_ with TMS as an internal standard. High-resolution mass spectra (HRMS) were obtained with a MALDI SYNAPT G2 HDMS system (Waters, Milford, MA, USA). In general, the designed probe-KCP compounds were synthesized as depicted in Scheme 1, and the experimental details are shown in Scheme 1.

### 3.2. Synthesis of KCP-OCH_3_

The synthesis of carbazole compound **2** was performed in accordance with methods described in the literature [36,37]. The synthesis of the compound KCP-OCH_3_ was also based on the literature [38]. Compound **2** (1 mmol) was added to a solution of 4′-methoxyacetophenone (1 mmol) in ethanol (10 mL), and fresh sodium ethoxide (4 mmol) solution (5 mL) was then added slowly. The mixture was stirred at room temperature overnight. The resulting precipitate was filtered and recrystallized from ethanol to give (*E*)-3-(9-ethyl-9*H*-carbazol-3-yl)-1-(4-methoxyphenyl)-prop-2-en-1-one (KCP-OCH_3_) as a faint yellow solid in 85% yield: ^1^H-NMR (500 MHz, CDCl_3_) *δ*_H_ 8.36 (s, 1H), 8.14 (d, *J* = 7.7 Hz, 1H), 8.10 (d, *J* = 8.5 Hz, 2H), 8.06 (d, *J* = 15.5 Hz, 1H), 7.77 (d, *J* = 8.4 Hz, 1H), 7.60 (d, *J* = 15.5 Hz, 1H), 7.50 (t, *J* = 7.6 Hz, 1H), 7.40 (dd, *J* = 13.6, 8.3 Hz, 2H), 7.29 (t, *J* = 7.4 Hz, 1H), 7.00 (d, *J* = 8.5 Hz, 2H), 4.34 (q, *J* = 7.1 Hz, 2H), 3.89 (s, 3H), 1.44 (t, *J* = 7.2 Hz, 3H); ^13^C-NMR (125 MHz, CDCl_3_) *δ*_C_ 188.73, 163.08, 145.52, 141.25, 140.36, 131.53, 130.62, 126.20, 126.00, 123.35, 122.78, 121.40, 120.56, 119.57, 118.69, 113.68, 108.79, 77.00, 55.40, 37.67, and 13.78. The HRMS (ESI) *m*/*z* was calculated for C_24_H_22_NO_2_^+^ [M + H]^+^: 356.1651, Found 356.1641.

### 3.3. Synthesis of the probe-KCP

Boron tribromide (as a 1.0 M solution in dichloromethane; 2 mL, 2 mmol) was added dropwise over a 10 min period to a suspension of KCP-OCH_3_ (0.5 mmol) in dry dichloromethane (15 mL cooled to 0 °C). The resulting mixture was stirred at 0 °C to room temperature. Water and dichloromethane were added. The organic layer was separated and dried with anhydrous Na_2_SO_4_. After concentrate under reduced pressure, the crude product was used for the next step, without separation or purification. Then, Cs_2_CO_3_ (Equation (2)) was added and stirred at room temperature for 15 min. Subsequently, 2,4-dinitrofluorobenzene was added and stirred at room temperature for 2 h. The resulting precipitate was filtered and recrystallized from ethyl acetate: petroleum ether (*v*:*v* = 2:8) to (*E*)-1-(4-(2,4-dinitrophenoxy)phenyl)-3-(9-ethyl-9*H*-carbazol-3-yl)prop-2-en-1-one (probe-KCP) as a give yellow solid (yield 76%): ^1^H-NMR (500 MHz, CDCl_3_) *δ*_H_ 8.84 (s, 1H), 8.43–8.28 (m, 2H), 8.22–8.02 (m, 4H), 7.78 (d, *J* = 8.3 Hz, 1H), 7.59–7.47 (m, 2H), 7.42 (t, *J* = 8.5 Hz, 2H), 7.32–7.18 (m, 3H), 7.12 (d, *J* = 9.0 Hz, 1H), 4.37 (d, *J* = 6.9 Hz, 2H), 1.45 (t, *J* = 6.8 Hz, 3H). ^13^C-NMR (125 MHz, CDCl_3_) *δ*_C_ 188.63, 156.93, 154.91, 147.21, 142.02, 141.55, 140.41, 136.66, 131.10, 128.85, 126.41, 125.54, 123.43, 122.70, 122.09, 121.75, 120.56, 119.83 (d, *J* = 11.5 Hz), 119.55, 118.10, 108.92, 77.00, 37.74, and 13.80. The HRMS (ESI) *m*/*z* was calculated for C_29_H_22_NO_2_^+^ [M + H]^+^: 508.1509, Found 508.1503.

### 3.4. Preparation of the Test Solutions

The fluorescence properties of the probe-KCP for thiophenols were evaluated in DMSO/PBS buffer (1:1, *v*/*v*, 20 mM, pH = 7.4). A stock solution of the probe-KCP was prepared at a concentration of 2.5 mM in 5 mL of DMSO and then diluted to the desired concentration with PBS (20 mM, pH = 7.4, containing 50% DMSO). Stock solutions of thiophenols and other analytes, in addition to those of common anions and metal ions, were prepared at a concentration of 5 mM in distilled water and then diluted to the desired concentrations with PBS (20 mM, pH = 7.4, containing 50% DMSO). Absorbance and fluorescence spectral data were recorded 30 min after the addition of the analytes. For fluorescence measurements, the excitation wavelength was 410 nm, and emissions were acquired from 450 nm to 800 nm.

### 3.5. General Procedure for Analysis

Stock solutions (100 μM) of thiophenols (4-toluenethiol, 4-fluorothiophenol, 4-methoxy- thiophenol, and 4-aminothiophenol) and other analytes, including glutathione (GSH), cysteine (Cys), and homocysteine (Hcy); alanine (Ala), serine (Ser), aniline (Ph-NH_2_), phenol (Ph-OH), sodium hydrosulfide (NaSH) and ethanethiol (C_2_H_5_SH), sodium chloride (NaCl), Ferrous chloride (FeCl_2_), Potassium chloride (KCl), calcium chloride (CaCl_2_), ferric chloride (FeCl_3_), magnesium sulfate (MgSO_4_), Sodium nitrate (NaNO_3_), Potassium fluoride (KF) and Sodium bicarbonate (NaHCO_3_) were prepared with PBS buffer (20 mM, pH = 7.4, containing 50% DMSO). The resulting solution was kept at 37 °C for 30 min before recording its absorption and fluorescence spectra.

### 3.6. Kinetic Study

To determine the reaction time of the probe-KCP, the increase in fluorescence intensity upon the addition of thiophenols (100 μM) to the probe solution (10 μM) in DMSO/PBS buffer (1:1, *v*/*v*, 20 mM, pH = 7.4) was investigated using a microplate reader. As shown in Figure S1, the addition of 4-methoxythiophenol immediately led to significant fluorescence enhancement at 540 nm for up to 30 min, followed by steady-state fluorescence. Taken together, these data indicated that the reaction time for the detection of thiophenols was 30 min. Thus, 30 min was considered an appropriate reaction time in subsequent experiments. The kinetic study indicated that the probe-KCP had a rapid response time, with high sensitivity for the detection of thiophenols.

### 3.7. Fluorescence Quantum Yield

The fluorescence quantum yield of the probe-KCP was determined according to methods described in the literature [39] using rhodamine 6G (*Φ* = 0.95, *λ*_ex_ = 530 nm, in ethanol solutions) as a fluorescence standard. The *u* values of the samples were calculated using the following equation:

(2) $$\phi_{u}=\frac{F_{u}}{Fs}\times \frac{A_{s}}{Au}\times \frac{n_{u}^{2}}{n_{s}^{2}}\times \phi_{s}$$

where the subscripts *u* and *s* denote the test and standard respectively, *Φ* is the fluorescence quantum yield, *A* is the absorbance at the excitation wavelength, *F* is the area under the corrected emission curve, and *n* is the refractive index of the solvent used. The subscripts *s* and *u* refer to the standard and the sample.

### 3.8. Reaction Mechanism

**Scheme 2:**
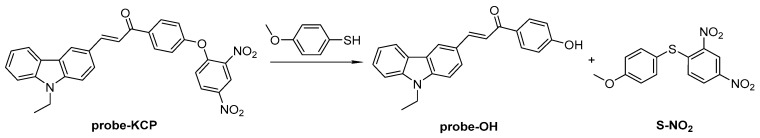
Proposed thiophenol detection mechanism of the probe.

A possible reaction mechanism of the probe-KCP with thiophenols was proposed based upon studies of the UV-vis spectra, the fluorescence intensity and other experiments. The reaction products were investigated by ^1^H-NMR, ^13^C-NMR, and mass spectrometry. The likely interaction mechanism of the probe-KCP with thiophenols is shown in Scheme 2. The probe-KCP (Equation (1)) and 4-methoxythiophenol (Equation (1)) was dissolved in the DMSO/PBS (20 mM, pH = 7.4) solution, followed by stirring at room temperature for 2 h. The mixture was diluted with 10 mL of water and extracted by ethyl acetate (3 × 10 mL). The organic layer was evaporated under reduced pressure and purified by silica gel column chromatography to give probe-OH and S-NO_2_. The characterization data for the probe-OH and S-NO_2_ are given in the Electronic Supplementary Information (Figure S1).

### 3.9. Detection of Thiophenols in Water Samples

Deionized water, artificial sea water, and tap water were filtered using a microfiltration membrane before use. The studied water samples were kept at 4 °C before use. The water samples were spiked with different concentrations of thiophenol (0, 2.0, 4.0, 6.0, 8.0, 10.0 μM). The solutions were incubated for 30 min before measurement and the fluorescence intensity was observed at 540 nm. 

### 3.10. Cell Culture

A human lung cancer cell line, A549, was purchased from the Cell Bank, Chinese Academy of Sciences (Shanghai, China). The cells were maintained in F-12K, supplemented with 10% FBS, 100 U/mL of penicillin, and 100 µg/mL of streptomycin at 37 °C. The cells were cultured at 37 °C in a humidified CO_2_ (5%) atmosphere. 

### 3.11. Determination of Cell Viability

Cell viability was determined using a 3-(4,5-dimethyl-2-thiazolyl)-2,5-diphenyl tetrazolium bromide (MTT) assay. Cells in growth phase were plated into a 96-well culture plate at a density of 3 × 10^3^ cells per well and incubated for 24 h. The cells were then exposed to 2.5, 5, 10, 20, and 50 µM of the probe-KCP for 24 h. Following this treatment, the cells were further incubated with MTT for 4 h. The medium in each well was carefully removed, and DMSO (150 μL) was then added to each well. The samples were thoroughly agitated for 10 min on a shaker. Finally, the absorbance of the samples at 490 nm was measured using a microplate reader. 

### 3.12. Fluorescence Microscopic Cell Imaging

The A549 cells were seeded on a cover glass and incubated overnight. The probe-KCP dissolved in DMSO was added to the cell medium at a final concentration of 10 µM for 30 min. The medium was then removed, and the cells were washed with PBS three times. For comparison purposes, the cells were pretreated with 4-methoxythiophenol (300 µM) at 37 °C for 30 min, followed by washing three times. Subsequently, the cells were treated with the probe-KCP for 30 min. The cells were imaged on a FV1000 confocal laser scanning microscope (CLSM, Olympus, Tokyo, Japan).

## 4. Conclusions

In summary, we described the synthesis of a safe and efficient chalcone fluorescent probe-KCP for the detection of thiophenols. The probe-KCP responded selectively to thiophenols over other amino acids and common metal ions and to potential interference from other substances in DMSO/PBS (1:1, *v*/*v*, 20 mM, pH = 7.4). The detection limit of the probe was 28 nM (R^2^ = 0.9946). Thus, we conclude that the probe-KCP exhibits high selectivity toward thiophenols. We used the probe to successfully monitor and quantify highly toxic thiophenols. This turn-on fluorescence probe features a remarkably large Stokes shift (130 nm) and a fast response time (30 min). It is highly selective and sensitive in terms of the detection of thiophenols, with significant fluorescence enhancement. In addition, this probe can be successfully applied to detect thiophenol in real water samples and in living cells. Therefore, this work provides a chalcone fluorescent probe (probe-KCP) for the detection of the highly toxic thiophenols found in environmental and biological samples.

## Figures and Tables

**Table 1:** Determination of thiophenol concentrations in water samples.

Sample	Thiophenol Spiked (μM)	Thiophenol Recovered (μM)	Recovery (%)
Artificial seawater	0	not detected	
	2	1.99 ± 0.334	100%
	4	3.79 ± 0.5484	95%
	6	5.63 ± 0.560	94%
	8	8.41 ± 0.617	105%
	10	10.12 ± 0.777	101%
Tap water	0	not detected	
	2	1.84 ± 0.105	92%
	4	4.19 ± 0.411	105%
	6	6.12 ± 0.397	102%
	8	8.57 ± 0.464	107%
	10	9.99 ± 0.405	100%
Deionized water	0	not detected	
	2	2.11 ± 0.304	106%
	4	3.80 ± 0.453	95%
	6	6.21 ± 0.3555	104%
	8	8.11 ± 0.605	101%
	10	9.62 ± 0.592	96%

## Supplementary Materials

The following are available online, Figure S1. date for investigation of the sensing mechanism. Figure S2. The effect of pH on the fluorescence intensity (*λ*_em_ = 540 nm) of probe-KCP (10 μM, *λ*_ex_ = 410 nm) in DMSO/PBS buffer (1:1, *v*/*v*, 20 mM) upon addition of 100 μM 4-methoxy thiophenol after incubation at 37 °C for 20 min. Figure S3. Fluorescence responses of probe-KCP (10 μM) to thiophenol and other various analytes (100 μM) in PBS buffer solution (20 mM, pH = 7.4) containing 50 % DMSO. Figure S4. Enhanced fluorescence response at 540 nm of the probe-KCP (10 μM) to thiophenol and other various analytes (100 μM) in PBS buffer solution (20 mM, pH = 7.4) containing 50 % DMSO. Figure S5. Percentage of viable A549 cells after treatment with different concentrations of the probe-KCP after 24 h using an MTT assay. Figure S6. (A)Time-dependent the fluorescence response of probe-KCP (10 μM) in the absence (blank) and presence of 4-methoxythiophenol, GSH, Cys, Hcy, NaSH or C_2_H_5_SH (10 equiv) in PBS buffer solution (20 mM, pH = 7.4) containing 50 % DMSO. (B) Time-dependent the fluorescence response of probe-KCP (10 μM) in the absence (blank) and presence of 4-methoxythiophenol, GSH, Cys, Hcy, NaSH or C_2_H_5_SH (10 equiv) at 0 min, 10 min, 20 min, 30 min. Figure S7. Time-dependent the fluorescence response of probe-KCP (10 μM) in the presence of 4-methoxythiophenol (10 equiv) in PBS/ DMSO solution (*v*:*v* = 1:1, 20 mM, pH = 7.4). Figure S8. Photograph of probe-KCP solutions (10 μM) in the presence of 4-methoxythiophenol (10 equiv) under natrual light and UV irradiation (365 nm).
