# Supplementary material for: Design and Synthesis of a Fluorescent Probe with a Large Stokes Shift for Detecting Thiophenols and Its Application in Water Samples and Living Cells
Source: Molecules. 2019 Jan 21;24(2):375. doi: 10.3390/molecules24020375 (PMC6359167; doi:10.3390/molecules24020375)
Supplement: Supplementary file 1 [file molecules-24-00375-s001.pdf]

Supporting Information (SI)

For

Design and synthesis of a fluorescent probe with a large Stokes shift for detecting thiophenols and its application in water sample and living cells

Hua Liu[a,b,c,d,1], Chuanlong Guo[a,e,1], Shuju Guo[a,b,c], Lijun Wang[a,b,c*]

and Dayong Shi[a,b,c,d *]

[a]CAS Key Laboratory of Experimental Marine Biology, Institute of Oceanology, Chinese Academy of Sciences, Qingdao 266071, China;

[b]Laboratory for Marine Drugs and Bioproducts, Qingdao National Laboratory for Marine Science and Technology, Qingdao 266237, China;

[c]Center for Ocean Mega-Science, Chinese Academy of Sciences, 7 Nanhai Road, Qingdao, 266071, P. R. China;

[d]University of Chinese Academy of Sciences, Beijing, China;

[e]Department of Pharmacy, College of Chemical Engineering, Qingdao University of Science and Technology, Qingdao 266042, China

* Correspondence: shidayong@qdio.ac.cn Tel: 86-532-82898719

* Correspondence: wanglijun@qdio.ac.cn Tel: 86-532-82898741

[1] These authors contributed equally to this work.

contents

1. Structure characterization S2

2. Additional spectra S5

1. Structure characterization

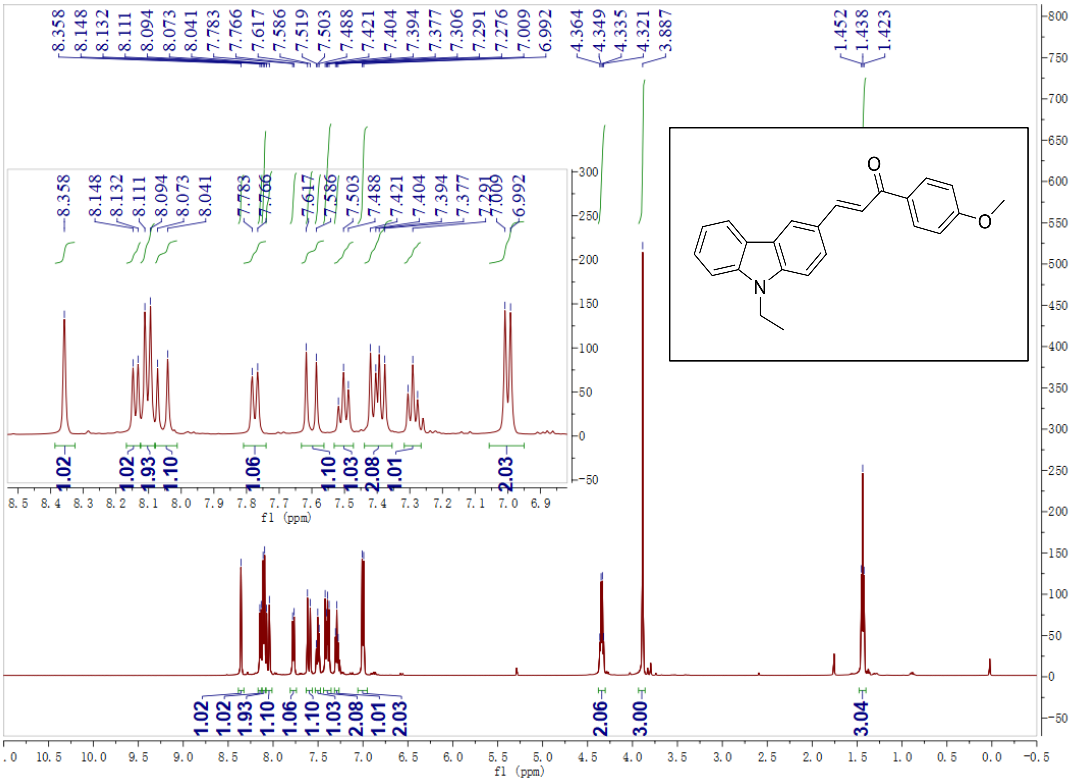

^{1}H NMR spectrum of KCP-OCH_3

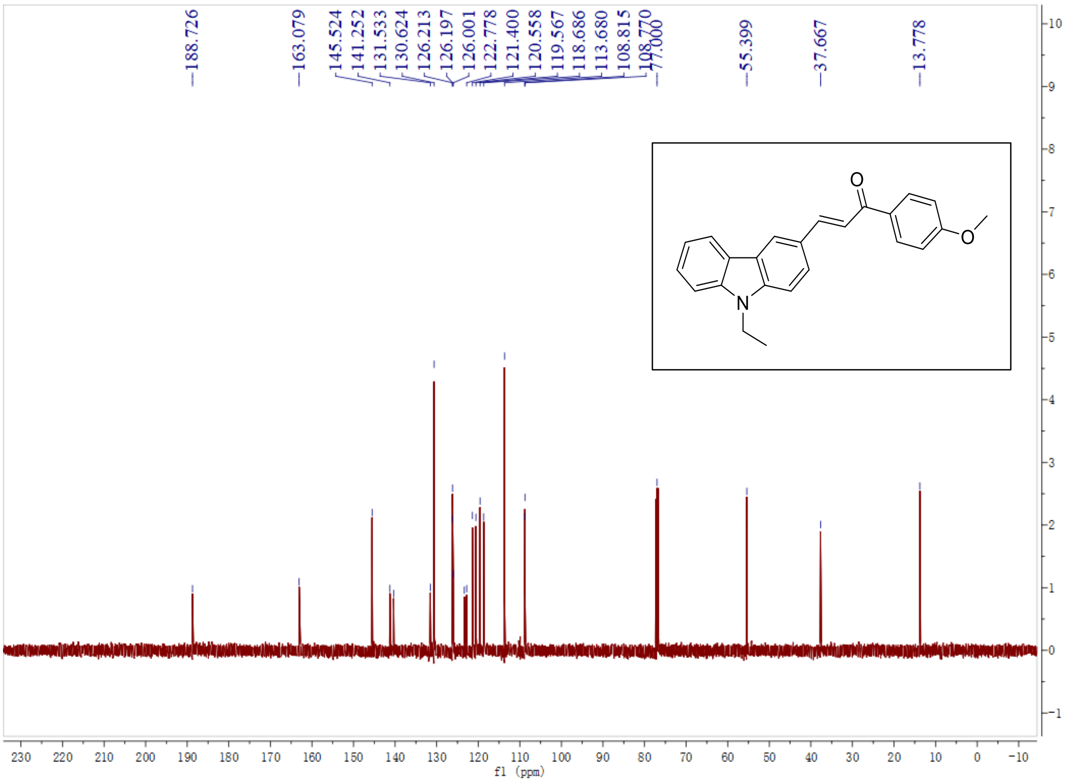

^{13}C NMR spectrum of KCP-OCH_3

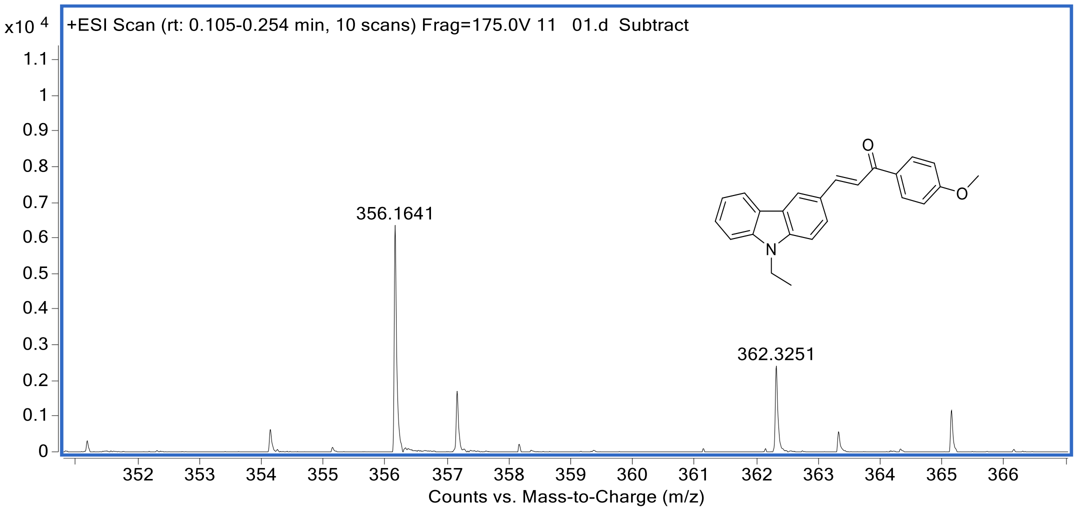

HR-MS spectrum of KCP-OCH_3

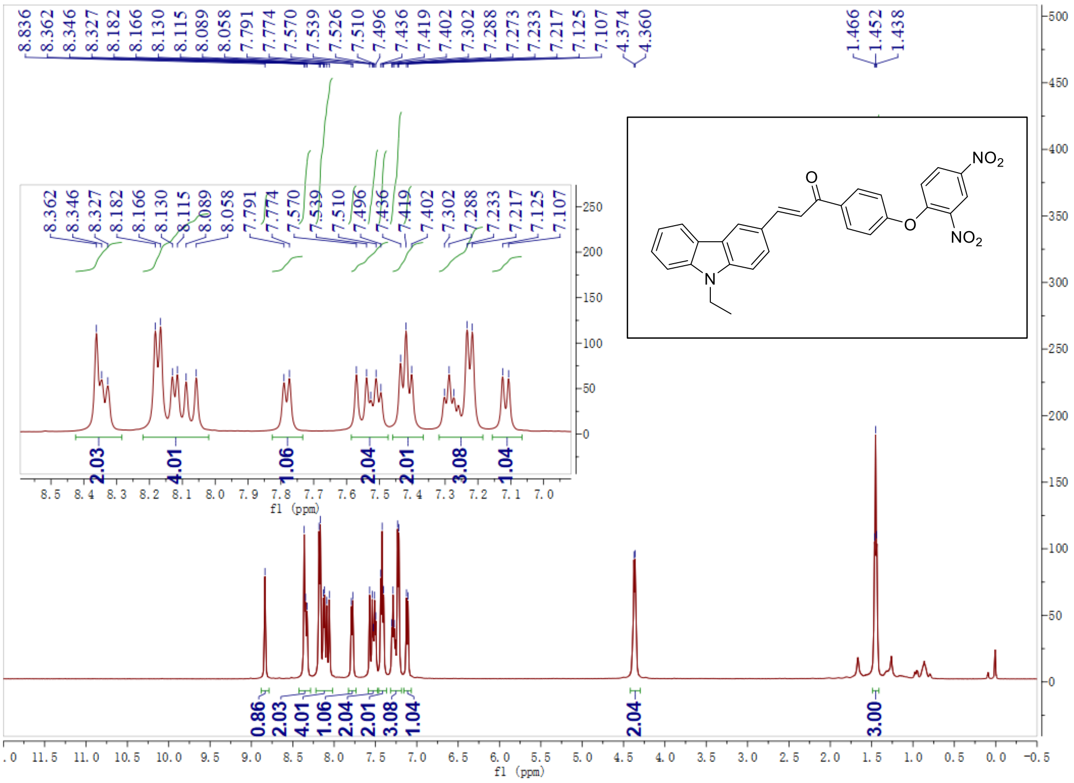

^{1}H NMR spectrum of probe-KCP

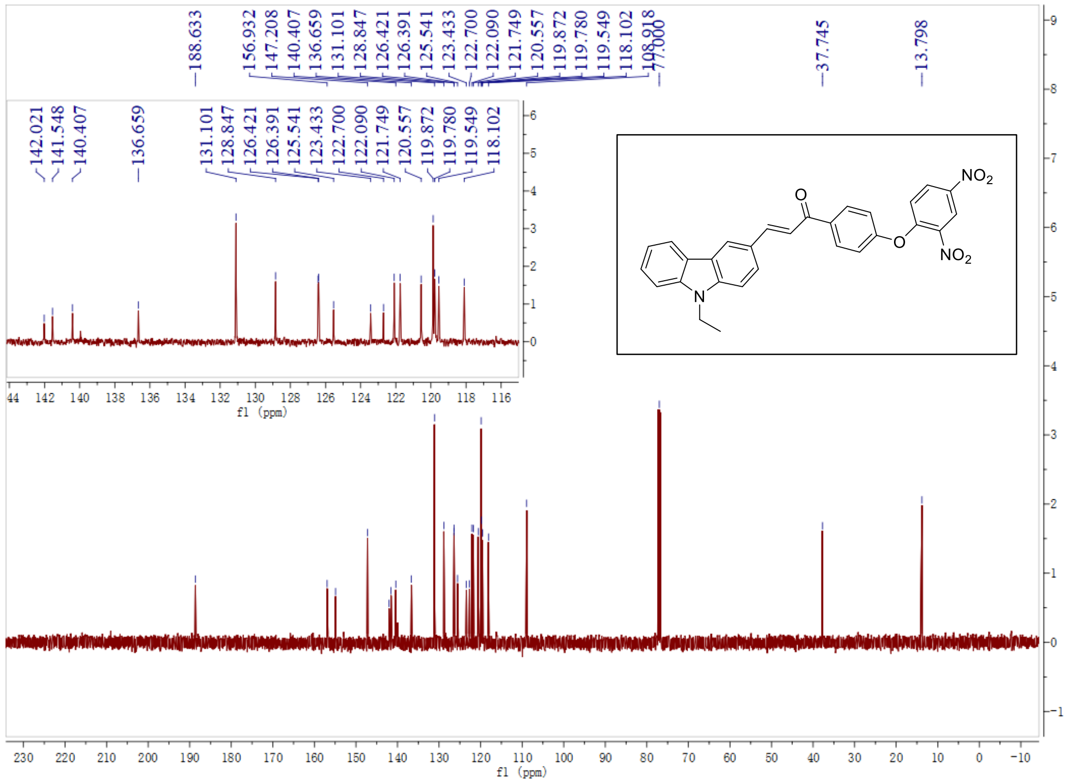

^{13}C NMR spectrum of probe-KCP

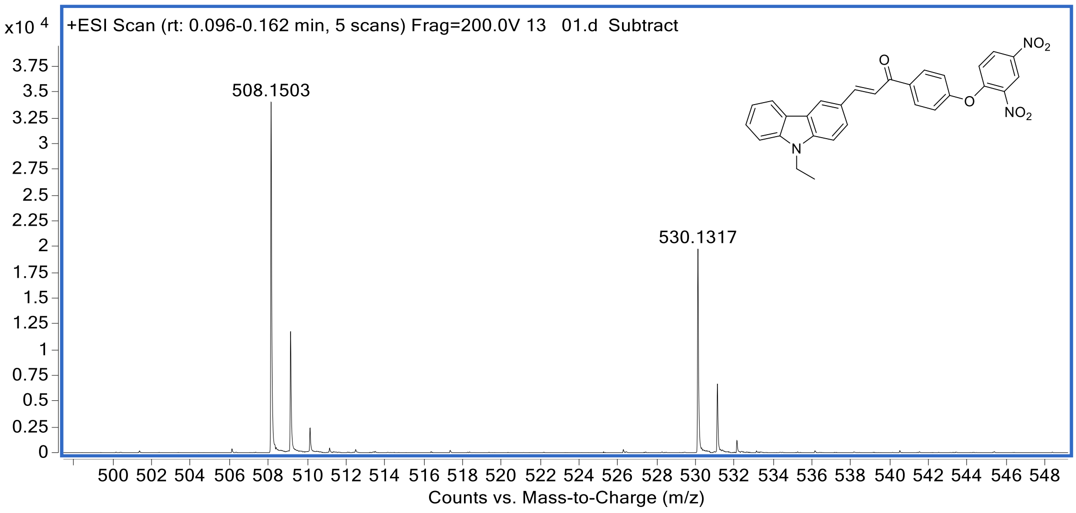

HR-MS spectrum of probe-KCP

2. Additional spectra

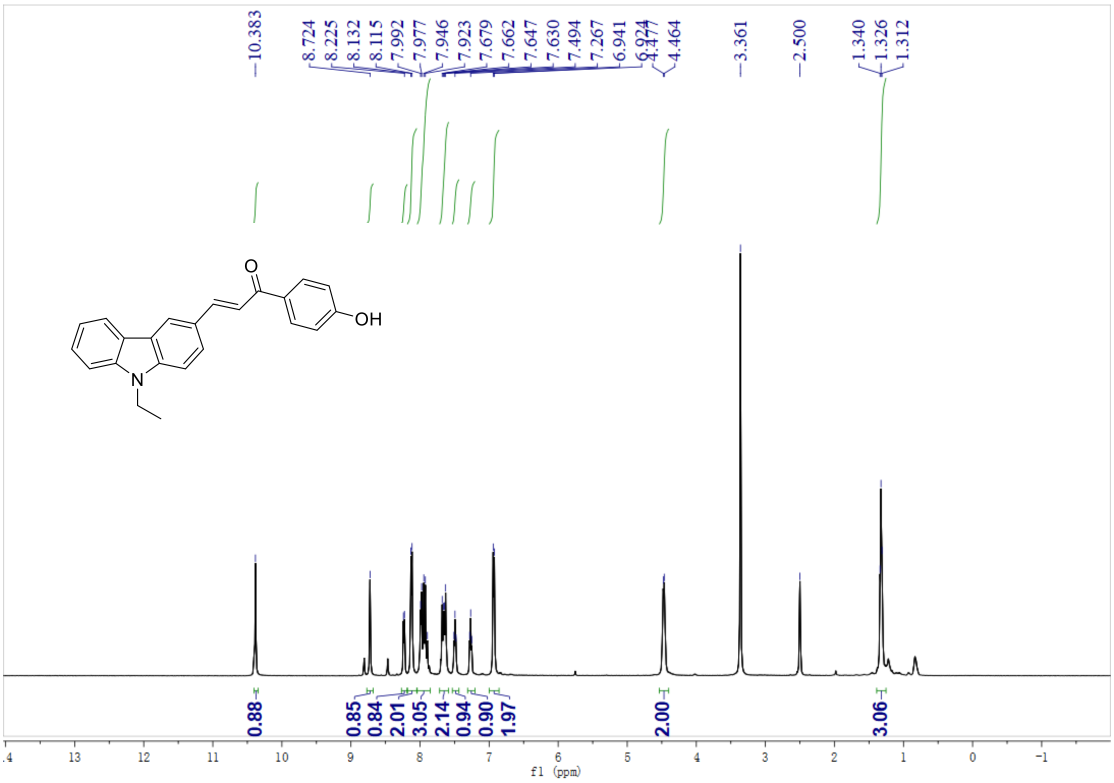

^{1}H NMR spectrum of probe-OH

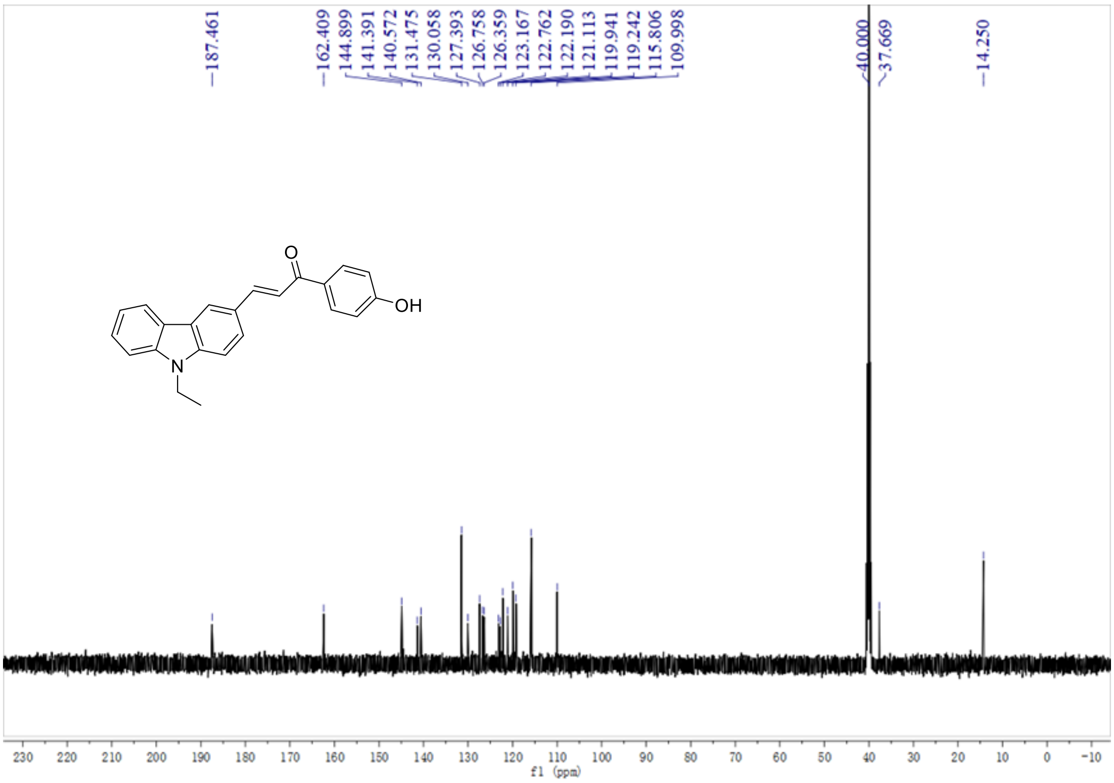

^{13}C NMR spectrum of probe-OH

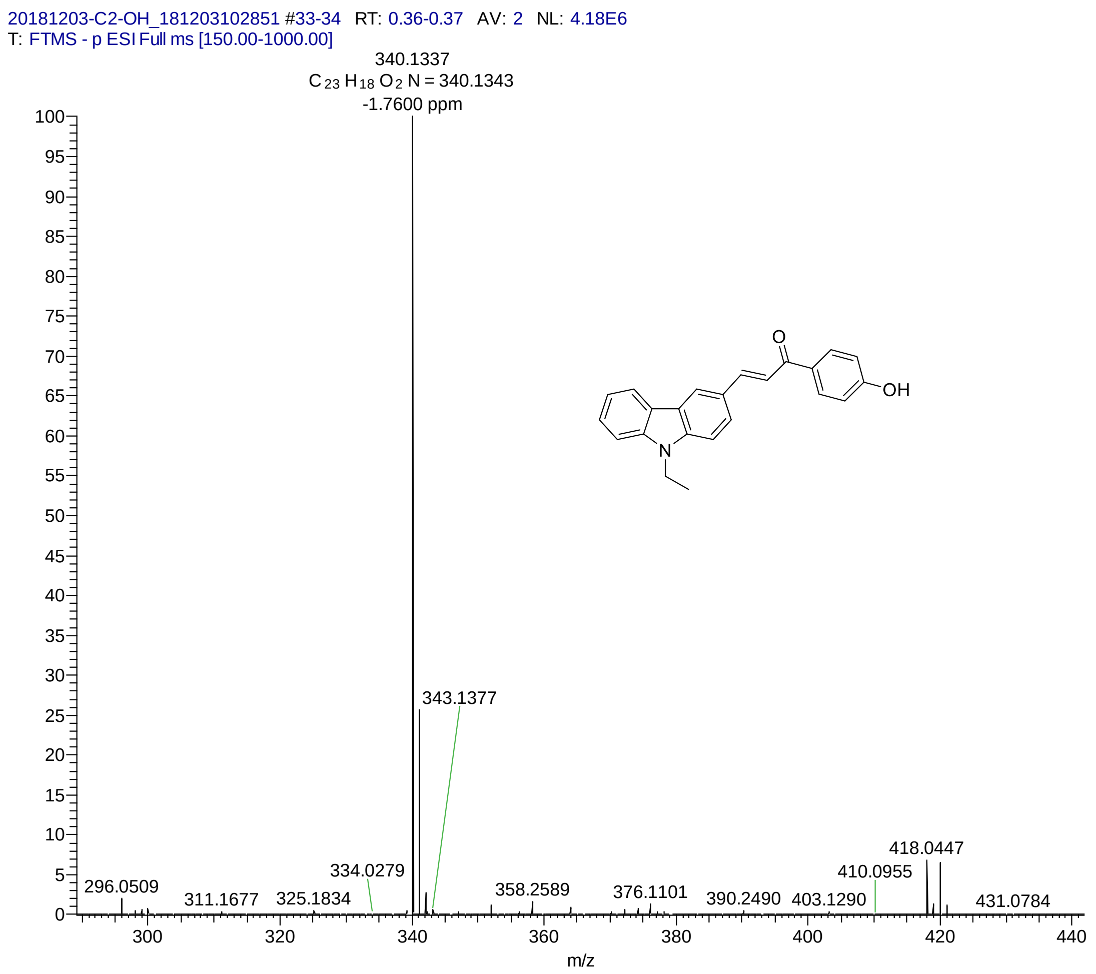

HR-MS spectrum of probe-KCN1

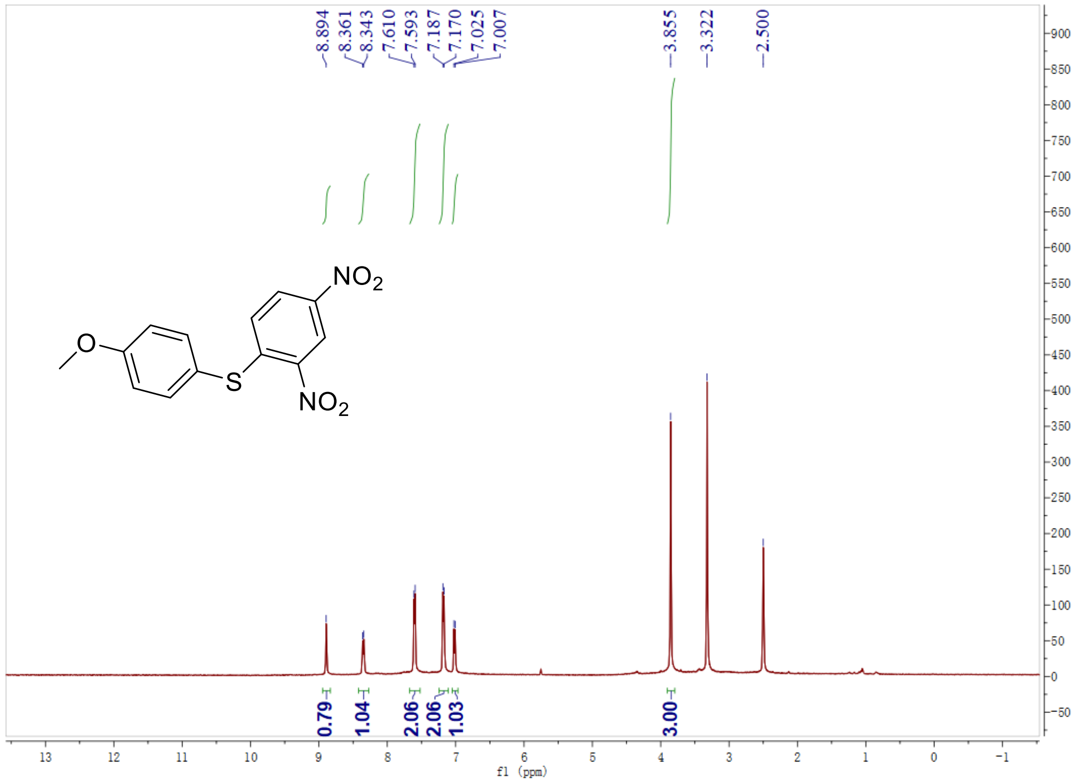

^{1}H NMR spectrum of S-NO_2

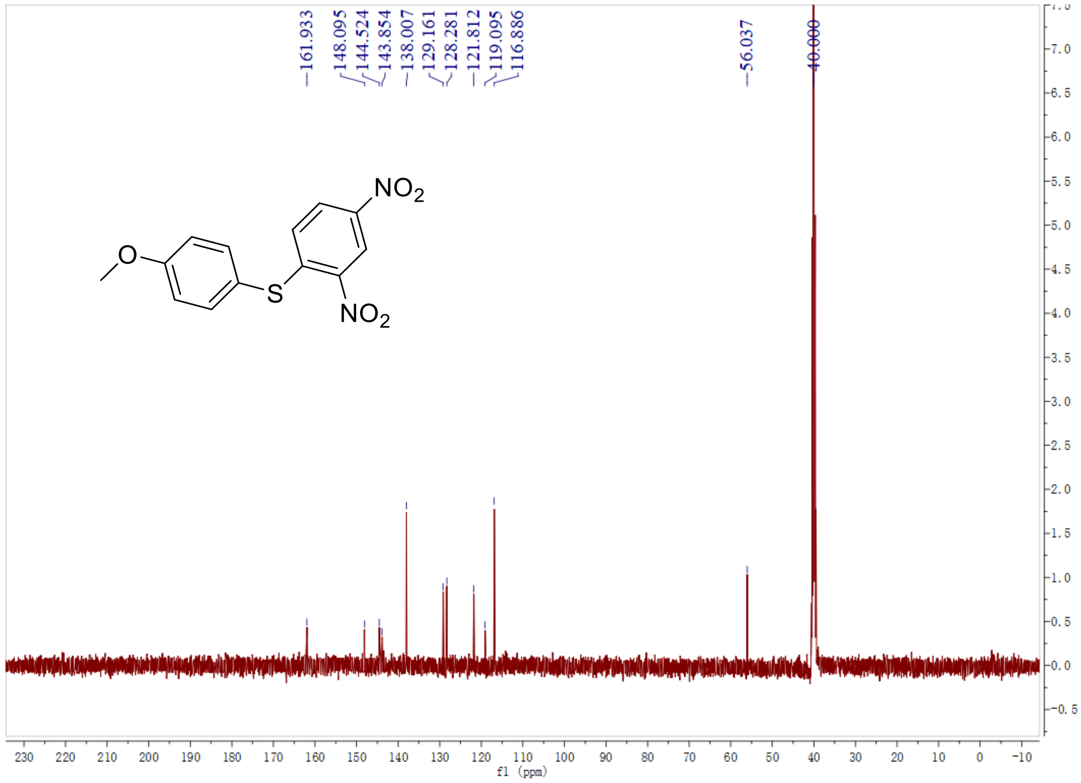

^{13}C NMR spectrum of S-NO_2

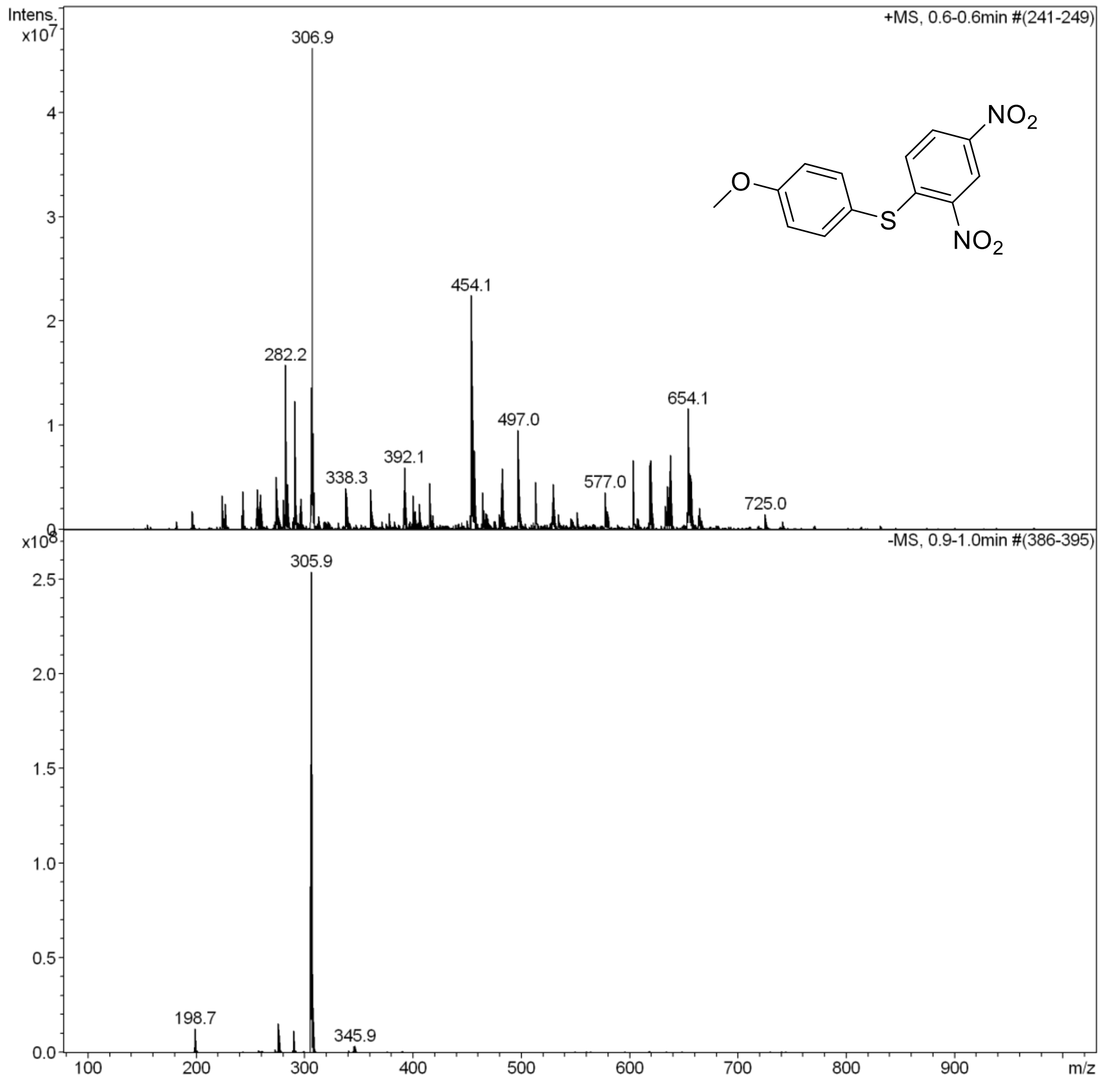

MS spectrum of S-NO_2

Figure S1. date for investigation of the sensing mechanism.

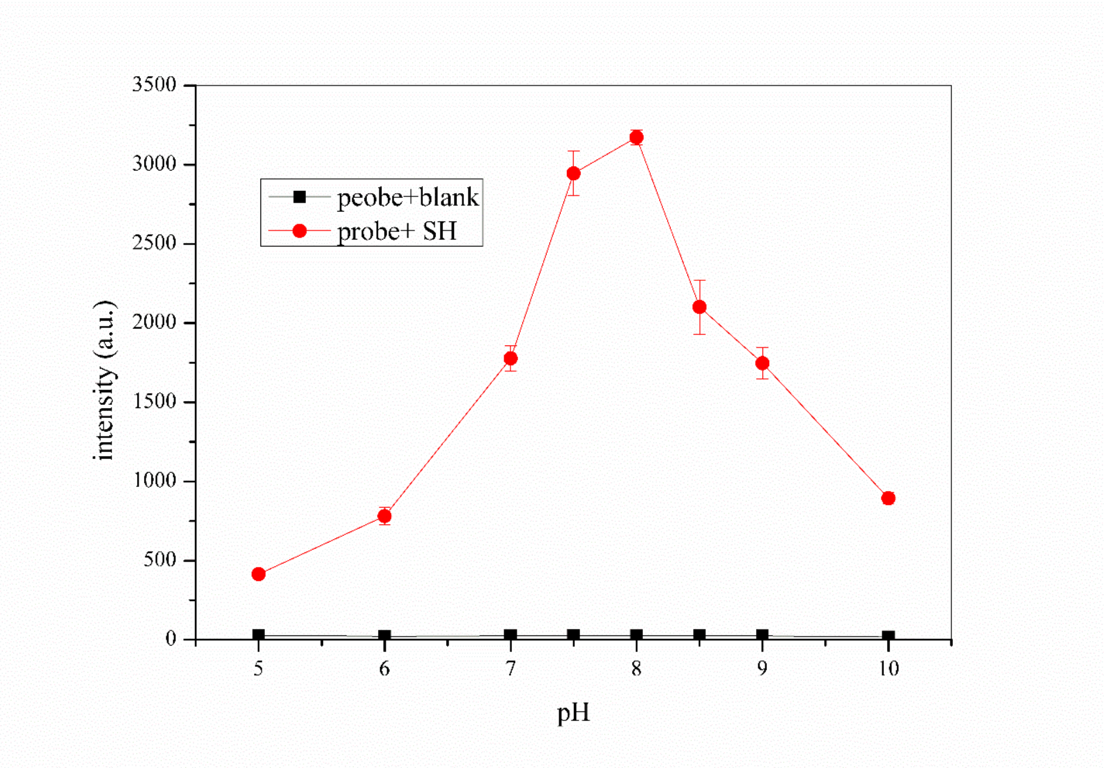

Figure S2. The effect of pH on the fluorescence intensity (λ_{em} = 540 nm) of probe-KCP (10 μM, λ_{ex} = 410 nm) in DMSO/PBS buffer (1:1, *v*/*v*, 20 mM) upon addition of 100 μM 4-Methoxy thiophenol after incubation at 37 ºC for 20 min.

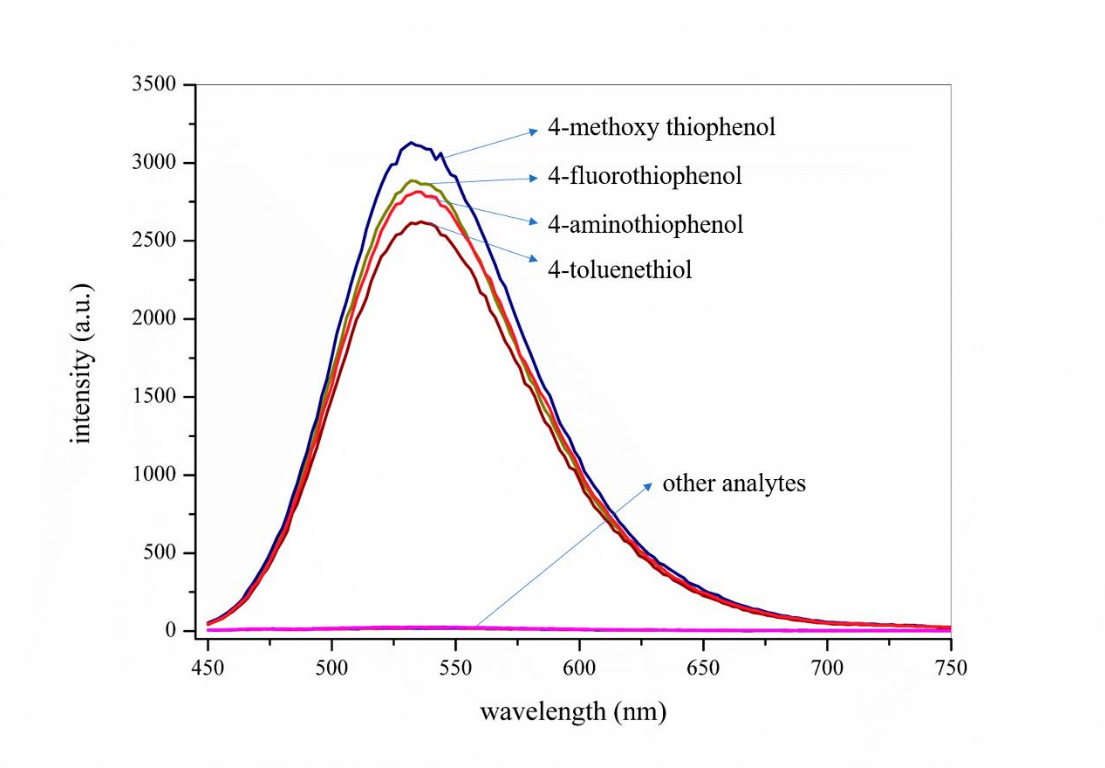

Figure S3. Fluorescence responses of probe-KCP (10 μM) to thiophenol and other

various analytes (100 μM) in PBS buffer solution (20 mM, pH = 7.4) containing 50 % DMSO.

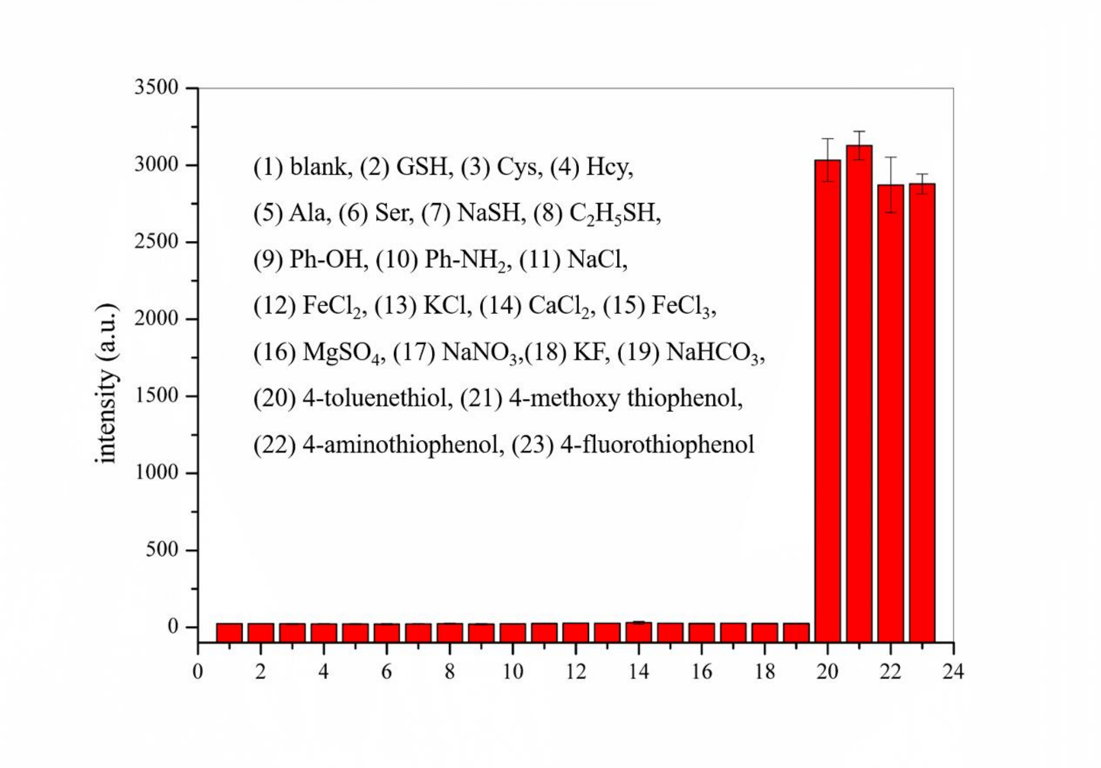

Figure S4. Enhanced fluorescence response at 540 nm of the probe-KCP (10 μM) to thiophenol and other various analytes (100 μM) in PBS buffer solution (20 mM, pH = 7.4) containing 50 % DMSO.

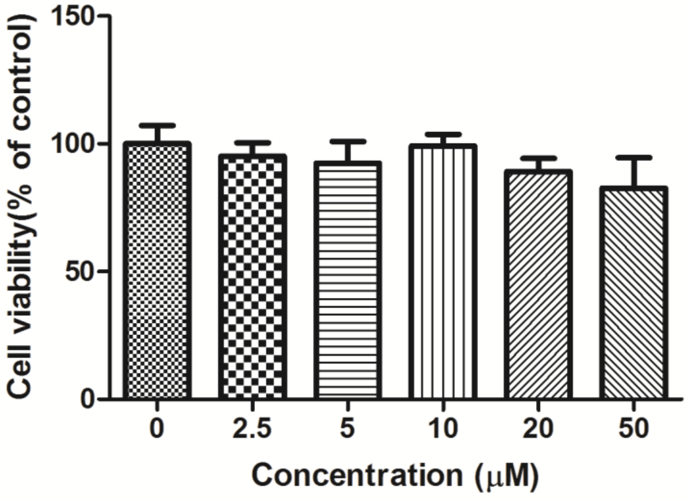

Figure S5. Percentage of viable A549 cells after treatment with different concentrations of the probe-KCP after 24 h using an MTT assay.

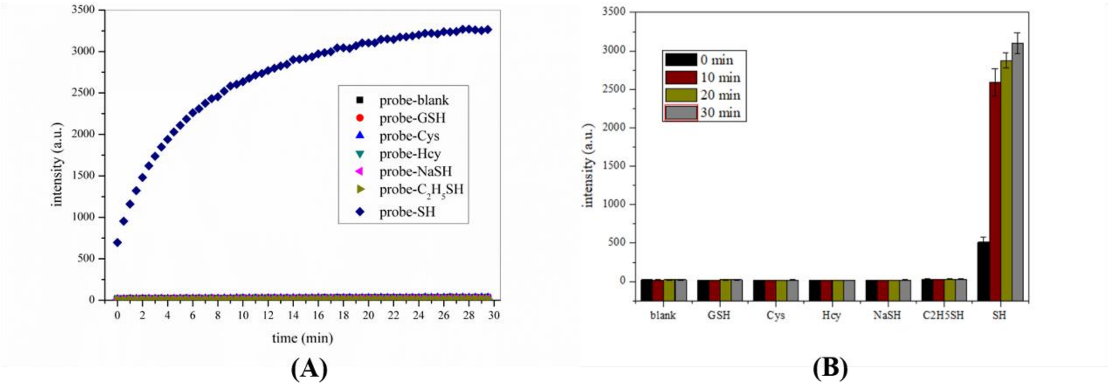

Figure S6. (A)Time-dependent the fluorescence response of probe-KCP (10 μM) in the absence (blank) and presence of 4-methoxythiophenol, GSH, Cys, Hcy, NaSH or C_2H_5SH (10 equiv) in PBS buffer solution (20 mM, pH = 7.4) containing 50 % DMSO. (B) Time-dependent the fluorescence response of probe-KCP (10 μM) in the absence (blank) and presence of 4-methoxythiophenol, GSH, Cys, Hcy, NaSH or C_2H_5SH (10 equiv) at 0 min, 10 min, 20 min, 30 min.

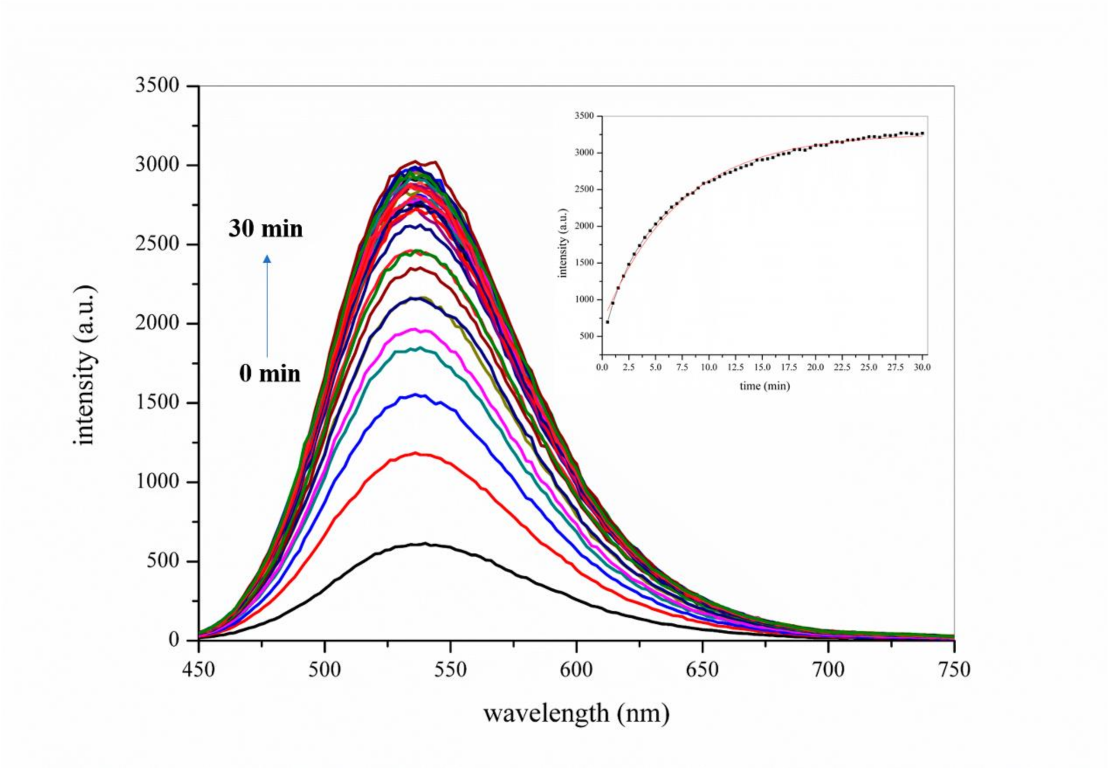

Figure S7. Time-dependent the fluorescence response of probe-KCP (10 μM) in the presence of 4-Methoxythiophenol (10 equiv) in PBS/ DMSO solution (v:v = 1:1, 20 mM, pH = 7.4).

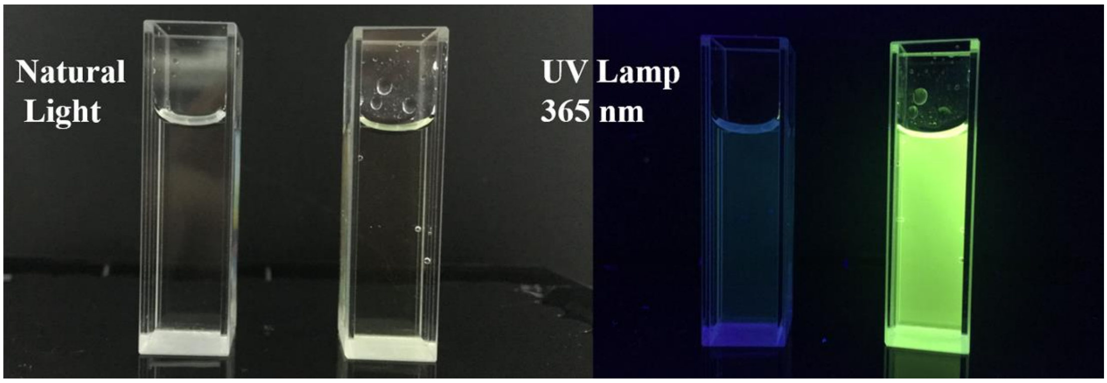

Figure S8. Photograph of probe-KCP solutions (10 μM) in the presence of 4-methoxythiophenol (10 equiv) under natrual light and UV irradiation (365 nm).

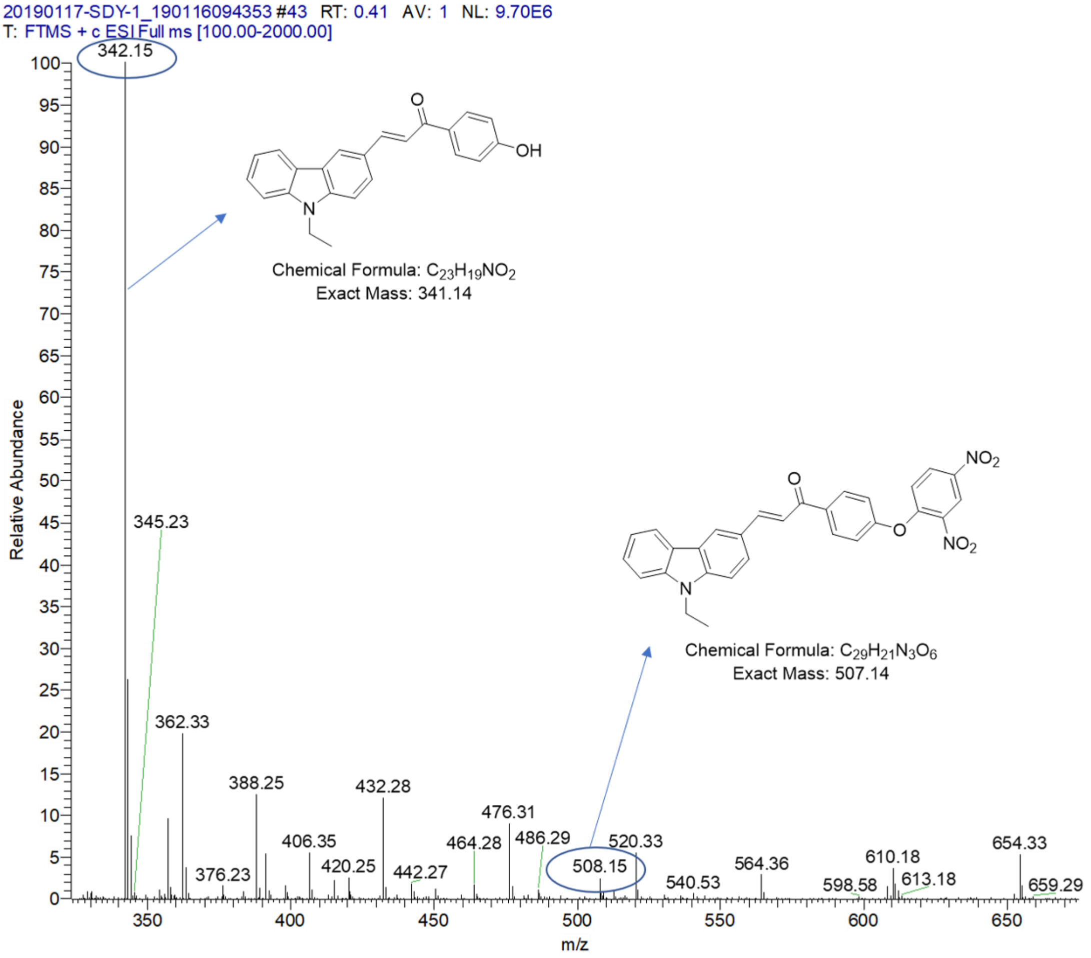

Figure S9. The ESI mass spectrum of probe-KCP in the presence of 4-methoxythiophenol.

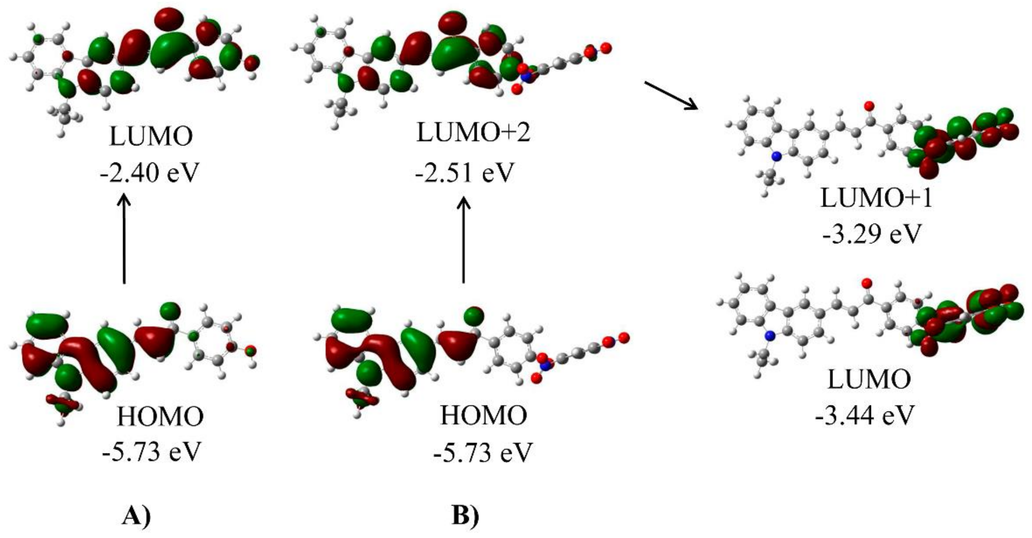

Figure S10. (A) Frontier molecular orbital plots of dye probe-OH in DMSO. (B) Frontier molecular orbital plots of probe-KCP in DMSO. The fluorescence emission of probe-KCN1 moieties is quenched by d-PET.
